# Preclinical study of reirradiation with hyperthermia in recurrent murine tumors and normal mouse skin

**DOI:** 10.2340/1651-226X.2025.43995

**Published:** 2025-07-27

**Authors:** Charlemagne A. Folefac, Priyanshu M. Sinha, Niels Bassler, Brita S. Sørensen, Michael R. Horsman

**Affiliations:** a Experimental Clinical Oncology-Department of Oncology; b Danish Center for Particle Therapy, Aarhus University Hospital, Aarhus, Denmark

**Keywords:** Re-irradiation, hyperthermia, recurrent tumor, tumor control, acute toxicity

## Abstract

**Background:**

Re-irradiation is an essential treatment option for recurrent tumours but is limited by normal tissue tolerance. Hyperthermia can enhance radiation efficacy by impairing DNA repair and improving tumor oxygenation; however, limited preclinical data are evaluating its combination with re-irradiation in recurrent tumor settings and normal skin.

**Objective:**

The study aims to determine optimal priming doses for skin and tumor response and evaluate the radiosensitising effect of hyperthermia when combined with re-irradiation in preclinical models.

**Methods:**

The right rear foot of non-tumor-bearing CDF1 mice or a C3H mammary carcinoma implanted in the foot were treated with a single radiation dose or reirradiation + hyperthermia (42.5°C, 1-h). Initial experiments identified a priming dose of 30 Gy that induced moderate but reversible acute skin toxicity and a tumor dose of 40 Gy that resulted in full regression with regrowth in 30–35 days from treatment. Reirradiation dose–response studies were conducted to determine the MDD₅₀ (skin) and TCD₅₀ (tumor) with and without hyperthermia. Thermal Enhancement Ratios (TER) and Therapeutic Gain Factor (TGF) were calculated.

**Results:**

The MDD₅₀ for reirradiation-induced skin damage was 25 Gy, reduced to 18 Gy with hyperthermia (TER = 1.4). In tumours, the TCD₅₀ decreased from 49 Gy (reirradiation alone) to 29 Gy with hyperthermia (TER = 1.7). A TGF of 1.2 was observed, indicating selective enhancement of tumor response relative to skin toxicity.

**Conclusion:**

Hyperthermia enhances the therapeutic effect of reirradiation by improving tumor control at lower doses, supporting its potential in recurrent cancer treatment strategies.

## Introduction

Recurrent tumours are difficult to treat, demanding refined therapeutic strategies. Re-irradiation, historically applied to brain, head and neck, and gynecological cancers, remains a key option when alternatives are limited [1]. Treating recurrent cancers remains challenging due to the heightened risk of normal tissue injury in anatomically sensitive regions [2–7]. Overcoming these challenges requires strategies that enhance the therapeutic ratio by increasing tumor control while sparing normal tissue.

Hyperthermia can enhance re-irradiation efficacy by improving tumor oxygenation and impairing DNA repair, thereby increasing radiation-induced cell death [8–11]. Hyperthermia also exerts direct cytotoxicity, particularly in tumor regions with low pH and poor microenvironmental conditions [12, 13]. The addition of hyperthermia to radiotherapy promotes necrosis [14], DAMP release (e.g. HSP70, HMGB1) [15], dendritic cell infiltration [16], and enhances NK cell activation [17] and cytotoxicity [18]. These effects position hyperthermia as a potent radiosensitising adjuvant.

Combining hyperthermia with re-irradiation may enable dose reduction while maintaining or improving tumor control, with clinical evidence supporting enhanced outcomes and reduced toxicity [19–28]. Moreover, a meta-analysis [29] and a systematic review [30] have further corroborated the benefits of this combined treatment approach.

Despite promising clinical outcomes, there is a notable gap in comprehensive preclinical data, particularly from recurrent tumor models, to inform the optimization of combined treatment strategies or support their ongoing use. To date, most preclinical reirradiation studies have primarily focused on healthy tissues or organs [31–38] and have not included a combination with hyperthermia or other treatments.

This study aims to optimize reirradiation combined with hyperthermia by using preclinical normal tissue and recurrent tumor models to enhance understanding of its therapeutic potential and refine strategies to maximize patient benefit in clinical practice.

## Material and methods

### Animal treatments

All experiments were conducted under the animal welfare policy of Aarhus University (http://dyrefaciliteter.au.dk) and approved by the Danish Animal Experiments Inspectorate (License number: 2021-15-0201-01008). Experiments used 10- to 14-week-old male CDF1 mice (Janvier Labs, Le Genest-Saint-Isle, France). The tumor model employed was the murine C3H mammary carcinoma, subcutaneously implanted in the right rear foot, as previously described [39].

Briefly, frozen tumor material was thawed and inoculated into the flank region every 3 months. Once tumors reached a suitable size, they were finely chopped under sterile conditions, and approximately 5–10 µL was injected into the right rear leg of each mouse. This location allowed access to the tumor while minimizing exposure to normal tissues and avoiding anesthesia during treatment. Mice were housed (up to four per cage) with free access to food and water under a 12-h light/dark cycle.

Tumors were treated when they reached ~200 mm³, calculated using: (length × width × height) × *π*/6. Mice with similarly sized tumors on the same day were redistributed into treatment groups. For normal tissue studies, animals were randomized. Body weight, physical appearance, feeding behavior, and movement were monitored weekly. Mice losing > 20% body weight or showing signs of illness were euthanized according to ethical guidelines.

### Radiation treatments

Irradiations were performed using a YXLON Maxishot X-ray unit operated at 280 kVp, equipped with a beryllium exit window and 1 mm copper added filtration. The measured half-value layer (HVL) was 2.6 mm Cu. The dose rate at the sample position was 2 Gy/min. Dosimetry was conducted using a Semifex ionization chamber (Type 31010, PTW, Germany), with calibration traceable to PTW Freiburg, in accordance with DIN 6809-4:2020-04. Mice were placed in a temperature-controlled water phantom (25 ± 1°C) with a Polymethyl methacrylate (PMMA) plate on top. Five mice were treated simultaneously in a setup similar to that previously described [40]. Animals were immobilized using lucite jigs without anesthesia. The tumor-bearing legs were fully submerged in the water and held in position by the use of tape loosely applied to the upper part of the leg. For acute skin toxicity, a drop of histoacrylic glue was applied near the upper leg and held with tape for 5 min for leg fixation. The tape was then loosened, and the animals rested for 10 min to restore circulation. This ensured no anesthesia was needed during irradiation.

### Hyperthermia treatments

Hyperthermia was performed using a circulating water bath (model TE 623; Heto, Birkerød, Denmark) at 42.5°C. Mice were immobilized in lucite jigs, and tumor-bearing legs were submerged in the bath as described for the radiation studies. The bath temperature was set 0.2°C higher than the target to match intratumoral temperature, based on previous studies [41, 42]. A certified mercury thermometer was used for temperature verification. Hyperthermia was applied for 1 h, beginning 30 min after reirradiation. This interval was selected because it is one that is generally used clinically [43]. Mice were monitored for up to 90 days post-treatment.

### Acute skin response

To select a prime dose for acute skin toxicity, non-tumor-bearing mice were irradiated with 26, 28, 30, 32, 34, or 36 Gy X-ray (*N* = 7–8 animals/group). A dose of 30 Gy was selected as optimal for initial treatment, as it caused a mild skin reaction (median toxicity score = 2), which improved to 1 after 30 days. This demonstrated an optimal balance between visible tissue damage and manageable side effects. For the re-Rt experiments, non-tumor-bearing mice received Re-Rt alone (20, 23, 25, 27, 30, 35 Gy) or Re-Rt + hyperthermia (15, 17.5, 20, 25 Gy), 30 days after the initial 30 Gy dose. Hyperthermia (42.5°C, 1 h) was initiated 30 min after reirradiation. A parallel single-dose experiment was also conducted by treating animals with 26, 28, 30, 32, 34, 36, 38 Gy (*N* = 6 animals/group). Skin toxicity was scored daily between days 8 and 28 from the day of re-Rt treatment, as described previously [44, 45]. Briefly, acute skin toxicity was assessed by visual inspection of the treated foot using a semi-quantitative scale (0.5–3.5, in 0.5 increments), capturing the extent of moist desquamation and tissue damage. Early signs included mild erythema and hair loss, progressing to swelling and localised desquamation. Severe reactions involved extensive skin loss, toe fusion, and deformation, culminating in complete desquamation and loss of foot structure. This scale enabled reliable, graded comparisons across treatment groups. The percentage of animals with a score ≥ 2.5 (moist desquamation) was used for the analysis.

### Tumor control response

To determine the optimal initial treatment dose for tumor control, a tumor regrowth assay was first performed. Tumor-bearing mice were treated with 0, 20, or 40 Gy X-rays when tumours reached approximately 200 mm³ (*N* = 5–11 animals/group). A single 40 Gy dose induced complete regression, with tumours regrowing to the initial volume within 30–35 days. Based on this, 40 Gy was selected as the priming dose for subsequent reirradiation studies. For the reirradiation experiments, tumours were again set up, allowed to grow to ~200 mm³, treated with 40 Gy, and monitored until they regressed beyond measurable size. Once tumours regrew to 200 mm³, mice were reirradiated with either single-dose Re-Rt alone (10, 15, 20, 25, 30, 35, 40, 45, 50, 55, and 60 Gy) or Re-RT combined with hyperthermia (5, 15, 25, 35, 45 Gy). In parallel, single-dose tumor control experiments were conducted using 35, 40, 45, 50, 55, 60, 65, 70 Gy (*N* = 3–8 animals/group). Tumor response was evaluated weekly over 90 days post-treatment. Tumor control was defined as the complete disappearance of the tumor from the irradiated leg, and the percentage of mice achieving control was recorded for each treatment group.

### Data analysis

Data were analyzed using GraphPad Prism. Non-linear regression was used to model tumor control and skin toxicity. For tumor control, the TCD50 (dose for 50% tumor control) was determined. For acute skin reactions, MDD_50_ (dose for 50% incidence of score ≥2.5) was calculated. The Thermal Enhancement Ratio (TER) was computed by dividing the MDD_50_ or TCD50 of Re-Rt alone by that of Re-Rt + hyperthermia. The therapeutic gain factor (TGF) was the ratio of the TER for the tumor to the TER for skin. Skin toxicity scores were plotted in Excel to display median values across treatment groups.

## Results

**Figure 1:**
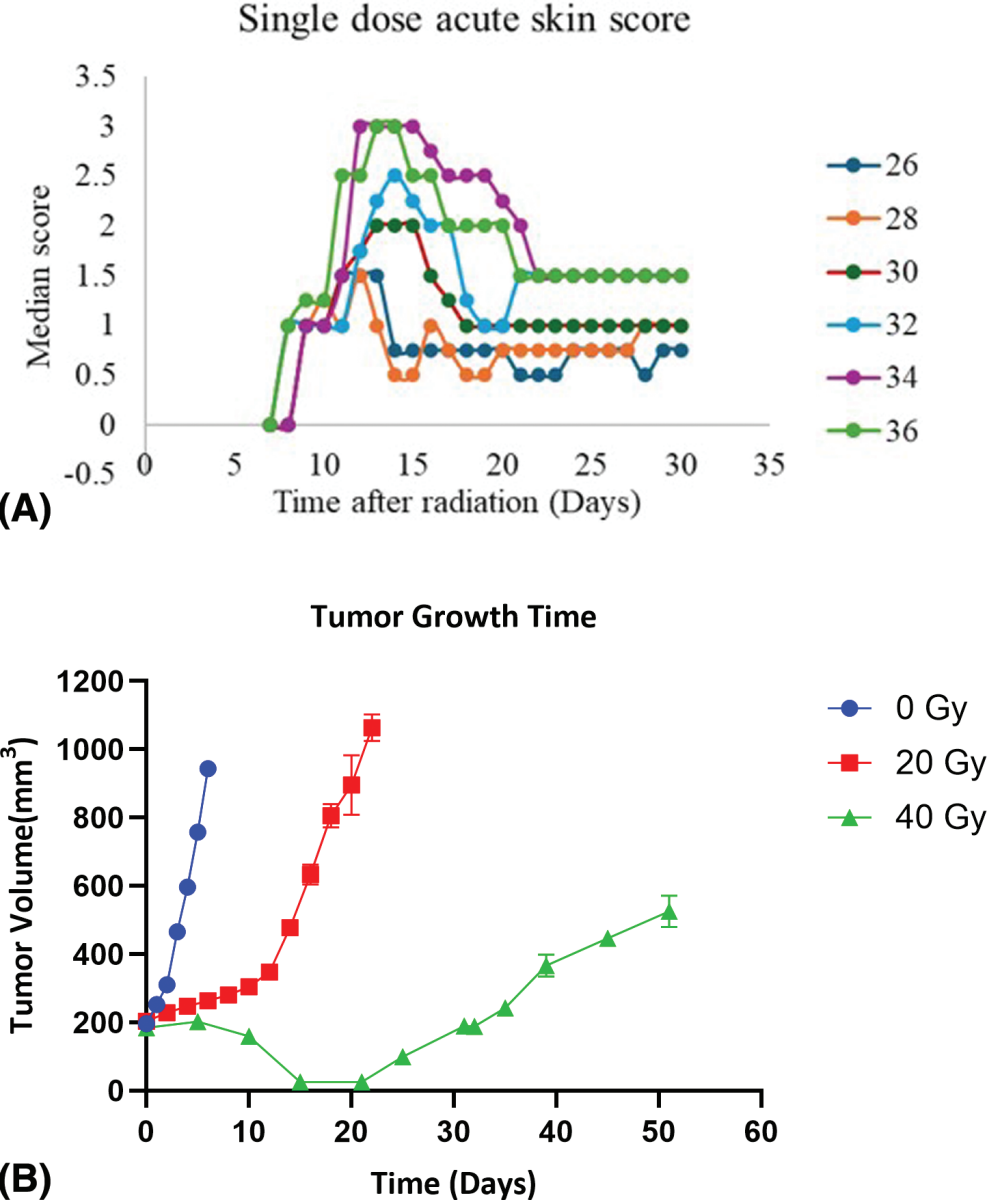
(A) Acute skin reaction after a single dose of X-ray photon irradiation treatment. While the y-axis shows the median acute skin reaction score, the X-axis shows the time after irradiation was administered. Results represent the median acute skin scores over 30 days following a single irradiation dose of 26–36 Gy. N = 7–8 animals per group. (B) Tumor growth time graph after treatment with 20 Gy and 40 Gy X-ray photon irradiation compared to control (0 Gy). The X-axis (Time [Days]) represents the number of days after treatment initiation, and the Y-axis (Tumor Volume) represents the tumor volume in mm3. Blue Circles (0 Gy), red squares (20 Gy), and green triangles (40 Gy). Results show mean values ± SED for 5–11 mice /group. Symbols without errors are where the errors are smaller than the symbols.

**Figure 2:**
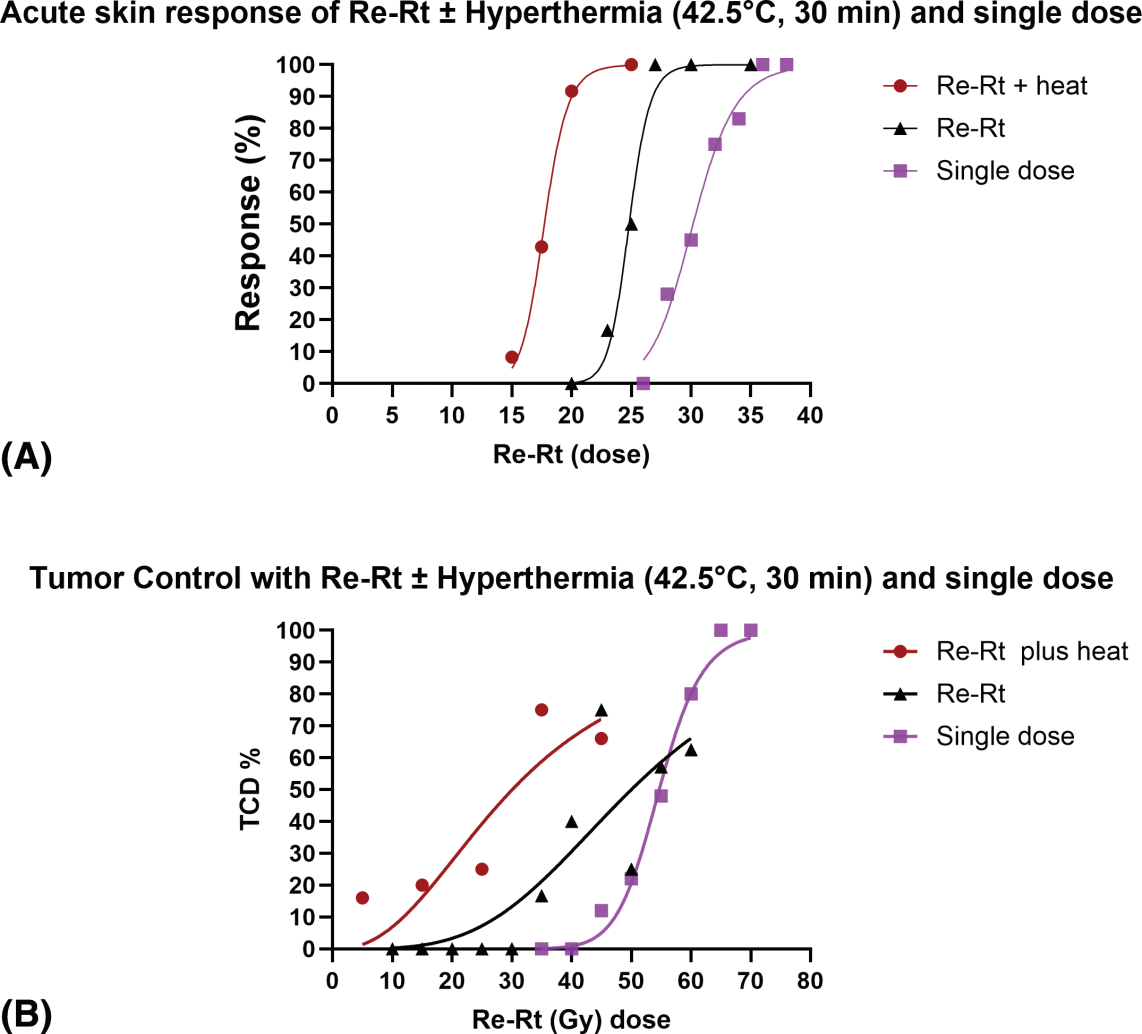
(A) Dose–response curves for acute skin reactions following single-dose irradiation, reirradiation alone, and reirradiation combined with hyperthermia. The graph shows the percentage of animals developing acute skin toxicity as a function of radiation dose. Hyperthermia at 42.5°C was administered for 1 h, starting 30 min after reirradiation. The treatment groups were: single-dose irradiation (purple squares; 26–36 Gy), reirradiation alone (black triangles; 25–35 Gy) delivered 30 days after an initial 30 Gy dose, and reirradiation combined with hyperthermia (red circles; 15–25 Gy). Each point represents the response rate within a dose group. *N* = 6 animals/group. (B) Tumor control probability following single-dose irradiation, reirradiation alone, and reirradiation combined with hyperthermia. The figure presents the percentage of tumor-bearing animals achieving local tumor control as a function of radiation dose. Hyperthermia at 42.5°C was applied for 1 h, beginning 30 min after reirradiation. The treatment groups were: single-dose irradiation (purple squares; 35–70 Gy), reirradiation alone (black triangles; 10–60 Gy), and reirradiation combined with hyperthermia (red circles; 5–45 Gy). Each point indicates the proportion of controlled tumors at the respective dose. *N* = 3–8 animals/group.

To systematically investigate the impact of reirradiation and hyperthermia on normal tissue and tumor response, we designed a series of studies with stepwise objectives. Our initial aim was to determine the dose-dependent effects of single-dose irradiation on acute skin toxicity in non-tumor-bearing mice. This provided a foundational understanding of normal tissue tolerance, identifying 30 Gy as a dose of interest that induced a moderate but reversible skin reaction (median skin score 2), which diminished over 30 days (Figure 1A). Next, we investigated the skin’s response to reirradiation, either alone or in combination with hyperthermia, following an initial 30 Gy dose. This experiment aimed to quantify the radiosensitising effect of heat on previously irradiated tissue. The reirradiation dose required to induce an acute skin reaction score ≥2.5 in 50% of animals (MDD_50_) was 25 Gy (95% CI: 23–26 Gy). When combined with hyperthermia (42.5°C, for 1 h starting 30 min after reirradiation), this threshold dropped to 18 Gy (95% CI: 10–31 Gy), corresponding to a TER of 1.4 (Figure 2A; Table 1). In comparison, the MDD₅₀ for acute skin damage following single-dose irradiation alone was estimated at 30 Gy (95% CI: 29–31 Gy), further contextualizing the sensitizing effect of reirradiation and hyperthermia.

**Table 1:** Summary of acute skin toxicity, tumor control, and therapeutic gain factor.

Treatment group	Skin MDD₅₀ (Gy, 95% CI)	Tumor TCD₅₀ (Gy, 95% CI)	Thermal enhancement ratio (TER)	Therapeutic gain factor (TGF)
Single-dose irradiation	30 (29–31)	54 (52–57)	–	–
Reirradiation alone	25 (23–26)	49 (41–59)	–	–
Reirradiation + hyperthermia	18 (10–31)	29 (19–46)	Skin: 1.4 Tumor: 1.7	1.2

TER: Re-Rt dose without hyperthermia divided by dose with hyperthermia for 50% effect. TGF: Tumor TER ÷ Skin TER. Dose values represent the radiation dose that causes a 50% incidence of an acute skin score of ≥ 2.5 or 50% tumor control, with 95% confidence intervals.

For the tumor control studies, the aim was to define the initial radiation dose required to induce complete tumor regression and subsequent regrowth. A single dose of 40 Gy induced full tumor regression, with regrowth evident between days 30 and 35. Lower doses, such as 20 Gy, resulted in delayed growth, while tumours in untreated controls continued to progress without regression (Figure 1B).

A single initial dose required for tumor control yielded a TCD₅₀ of 54.00 Gy (95% CI: 52–57 Gy) (Table 1; Figure 2B). In reirradiation studies, tumours initially treated with 40 Gy were retreated following regrowth, which took approximately 15–19 days post-regression to become palpable and reach the treatment volume of ~200 mm³. Reirradiation alone resulted in a TCD₅₀ of 49 Gy (95% CI: 41–59 Gy), while the addition of hyperthermia significantly reduced the TCD₅₀ to 29 Gy (95% CI: 19–46 Gy), corresponding to a TER of 1.7 (Figure 2B; Table 1). Comparison of tumor and skin TERs yielded a TGF of 1.2.

In the tumor control studies, 23 of 47 animals (48.9%) in the reirradiation plus hyperthermia group did not complete the study due to treatment-related morbidity. Of these, 6 were found dead in their cages, 13 were euthanized due to severe skin reactions or weight loss exceeding 20%, and 6 developed secondary tumours (Table 2). In contrast, substantially fewer toxicity-related deaths occurred in animals receiving reirradiation alone, despite being treated within a similar dose range associated with the adverse effects observed in the hyperthermia group. Due to this dose-limiting toxicity, a complete dose–response curve for reirradiation ± hyperthermia could not be achieved at higher doses.

**Table 2:** Animals that were lost in the heat experiment group and the reason.

Found death in the box	Severe skin damage or > 20% weight loss	Developed a secondary tumor
6	13	6

A total of 47 mice were included in the reirradiation plus hyperthermia group. Of these, 25 animals (53.2%) did not complete the study, with 19 mice (40.4%) excluded due to heat-associated toxicity, including death or euthanasia from severe skin reactions or significant weight loss.

## Discussion

In the current study, reirradiation effects with or without hyperthermia were systematically investigated in both healthy tissue and recurrent tumor models. The overall aim was to assess whether therapeutic efficacy could be enhanced without compromising normal tissue integrity – an area largely underrepresented in preclinical studies despite its clinical relevance [19–28].

To establish baseline normal tissue response, we first evaluated acute skin toxicity after a single 30 Gy dose, which induced a moderate but reversible reaction (median score 2) resolving by day 30. Reirradiation after 30 days required 25 Gy to induce a ≥2.5 skin score in 50% of mice (MDD₅₀), indicating partial recovery. This aligns with the findings of Terry et al. [46], who showed that murine skin retains a radiation ‘memory’ within the first month, with full recovery occurring between 2 and 6 months. Our findings reinforce the importance of optimizing inter-treatment intervals to improve normal tissue tolerance. Adding hyperthermia reduced the MDD₅₀ to 18 Gy (TER = 1.38), demonstrating enhanced radiosensitization in normal tissue.

In tumor-bearing mice, 40 Gy induced full regression with regrowth by day 30–35. Reirradiation alone resulted in a TCD₅₀ of 49 Gy, while the addition of hyperthermia reduced it to 29 Gy (TER = 1.69), yielding a TGF of 1.22. This suggests a favorable therapeutic window. Notably, the TCD₅₀ after single-dose irradiation was 54 Gy, highlighting the enhanced efficacy of reirradiation ± hyperthermia (Table 1; Figure 2B). To our knowledge, this is the first preclinical study showing a higher TER in tumours than in surrounding normal tissue after reirradiation plus hyperthermia, contrasting prior studies that focused on single radiation and heat treatments and normal tissue limits [31–36].

Given that partial recovery was observed 30 days post-irradiation in our normal tissue study, full recovery could likely be achieved at the current dose levels if the animals were monitored over a longer period based on data from previous studies. Additionally, the inter-treatment interval affects late toxicity [36]; therefore, future studies should assess different reirradiation schedules and include fractionation. Interindividual radiosensitivity also warrants consideration [2], supporting personalized reirradiation dosimetry. As reirradiation followed prior exposure (30 Gy skin, 40 Gy tumor), reduced MDD₅₀ and TCD₅₀ values likely reflect cumulative damage in the skin study rather than intrinsic radiosensitivity.

Interestingly, tumor volumes at the start of reirradiation matched those used in the single-dose experiments, suggesting that recurrent tumours may respond differently to a second insult. This could imply increased radiosensitivity in relapsed tumours; however, further studies are needed to investigate this, particularly in light of the microenvironmental and molecular changes that characterize tumor recurrence. Moreover, in our study, 48.9% of animals receiving hyperthermia plus reirradiation were excluded due to morbidity or secondary tumours, limiting curve completion (Table 2).

Clinically, reirradiation outcomes depend on tumor volume, tissue sensitivity, dose limits, and timing. Late effects such as fibrosis and organ dysfunction remain a challenge. While Intensity-Modulated Radiation Therapy (IMRT) and hyperfractionation reduce normal tissue exposure [37, 47], our data suggest that combining hyperthermia with reirradiation may lower required doses and preserve efficacy. However, narrow safety margins highlight the need for precise thermal dosimetry and toxicity monitoring in future clinical applications.

## Conclusion

This study provides preclinical evidence that hyperthermia enhances the efficacy of reirradiation to a greater extent in tumours than in normal tissue, indicating a potential therapeutic gain. However, associated toxicities highlight the need for dose optimization, incorporation of additional endpoints, diverse tumor models, and fractionated regimens in future studies. While the radiosensitising effect of hyperthermia is evident, its underlying mechanisms remain unclear. Elucidating the molecular and microenvironmental responses to hyperthermia in reirradiated tumours will be essential. Moreover, integrating hyperthermia with other modalities, such as immunotherapy, may further enhance tumor control while limiting normal tissue damage, supporting the development of more effective and individualized reirradiation strategies.

## Data Availability

Data presented in this study are available on request from the corresponding author. The data are not publicly available because the results from these experiments and all other animal experiments at our institute are stored in a single data depository; therefore, access is limited to relevant qualified personnel only.
